# 164. Performance of ePlex® Blood Culture Identification Panels in Clinical Isolates and Characterization of Antimicrobial Stewardship Opportunities

**DOI:** 10.1093/ofid/ofad500.237

**Published:** 2023-11-27

**Authors:** Jenni Thomas, Justin A Clark, Vaneet Arora, David Burgess, Donna R Burgess, Ryan Mynatt, Jeremy VanHoose, Katie L Wallace, Sarah Cotner

**Affiliations:** University of Virginia Health, Charlottesville, Virginia; University of Kentucky College of Pharmacy, 4161 Victoria Way, 16202, Kentucky; University of Kentucky, Lexington, KY; UK HealthCare, Lexington, KY; UK HealthCare, Lexington, KY; University of Kentucky Healthcare, Lexington, Kentucky; UK HealthCare, Lexington, KY; University of Kentucky HealthCare, Lexington, KY; UK HealthCare, Lexington, KY

## Abstract

**Background:**

Rapid diagnostic testing allows pathogen identification within hours of a positive blood culture. GenMark’s ePlex® Blood Culture Identification (BCID) Panels detect bacteria, fungi, and antibiotic resistance genes. This study assessed concordance of ePlex® organism identification with standard microbiologic identification methods and concordance of ePlex® genotypic resistance mechanism detection and standard susceptibility testing. Secondary outcomes included panel specific performance and characterization of antimicrobial stewardship opportunities.

**Methods:**

This retrospective, single center study included patients with a positive blood culture from May 2020 – January 2021. Polymicrobial cultures were excluded. Identification concordance was evaluated between ePlex® and MALDI-TOF, BD Phoenix™, and biochemical tests. Resistance mechanism/phenotypic susceptibility concordance was evaluated between ePlex® and BD Phoenix^TM^/ETEST®. MecA was assessed via oxacillin resistance, vanA via vancomycin resistance, CTX-M via ceftriaxone resistance, and KPC via carbapenem resistance. Opportunities for antimicrobial stewardship were also quantified.

**Results:**

The overall identification concordance rate in 1276 positive blood cultures was 98.2%, with rates of 98.5%, 98.1%, and 93.3% for Gram-positive, Gram-negative, and fungal isolates, respectively. In 381 staphylococcal isolates, concordance rates were 98.3% and 100% for the presence and absence of mecA. Both the presence and absence of vanA were 100% concordant in 63 enterococcal isolates. CTX-M and KPC were evaluated in 189 Enterobacterales. Concordance rates were 96.8% for the presence of CTX-M and 60% for the presence of KPC, demonstrating that 40% of carbapenem resistance was due to non-enzymatic mechanisms. Ceftriaxone and carbapenem susceptibility correlated with the absence of CTX-M and KPC, respectively, in 100% of cultures. A majority of ePlex results (69.5%) represented opportunities for potential antimicrobial stewardship intervention.

**Conclusion:**

High concordance rates between ePlex® and standard identification and susceptibility testing methods enable rapid antimicrobial optimization. Numerous opportunities for antimicrobial stewardship were identified based on ePlex® results.

**Disclosures:**

**All Authors**: No reported disclosures

